# E2/ER β Enhances Calcineurin Protein Degradation and PI3K/Akt/MDM2 Signal Transduction to Inhibit ISO-Induced Myocardial Cell Apoptosis

**DOI:** 10.3390/ijms18040892

**Published:** 2017-04-24

**Authors:** Kuan-Ho Lin, Wei-Wen Kuo, Marthandam Asokan Shibu, Cecilia-Hsuan Day, You-Liang Hsieh, Li-Chin Chung, Ray-Jade Chen, Su-Ying Wen, Vijaya Padma Viswanadha, Chih-Yang Huang

**Affiliations:** 1 College of Medicine, China Medical University, Taichung 40402, Taiwan; linalpra@hotmail.com; 2 Department of Emergency Medicine, China Medical University Hospital, Taichung 40402, Taiwan; 3 Department of Biological Science and Technology, China Medical University, Taichung 40402, Taiwan; wwkuo@mail.cmu.edu.tw; 4 Graduate Institute of Basic Medical Science, China Medical University, Taichung 40402, Taiwan; shibu.m.a@gmail.com; 5 Department of Nursing, Meiho University, Pingtung 900, Taiwan; x00003023@mail.meiho.edu.tw; 6 Department of Health and Nutrition Biotechnology, Asia University, Taichung 40402, Taiwan; hshsieh@asia.edu.tw; 7 Department of Hospital and Health Care Administration, Chia Nan University of Pharmacy & Science, Tainan County 700, Taiwan; jerlin15@yahoo.co.in; 8 Department of Surgery, School of Medicine, College of Medicine, Taipei Medical University, Taipei 220, Taiwan; rayjchen@tmu.edu.tw; 9 Department of Dermatology, Taipei City Hospital, Renai Branch, Taipei 220, Taiwan; suyinwen@ms15.hinet.net; 10 Department of Biotechnology, Bharathiar University, Coimbatore 641 046, India; padma.vijaya@gmail.com; 11 Graduate Institute of Chinese Medical Science, China Medical University, Taichung 40402, Taiwan

**Keywords:** 17β-Estradiol, calcineurin, cardiac apoptosis, isoproterenol, apoptosis

## Abstract

Secretion of multifunctional estrogen and its receptor has been widely considered as the reason for markedly higher frequency of heart disease in men than in women. 17β-Estradiol (E2), for instance, has been reported to prevent development of cardiac apoptosis via activation of estrogen receptors (ERs). In addition, protein phosphatase such as protein phosphatase 1 (PP1) and calcineurin (PP2B) are also involved in cardiac hypertrophy and cell apoptosis signaling. However, the mechanism by which E2/ERβ suppresses apoptosis is not fully understood, and the role of protein phosphatase in E2/ERβ action also needs further investigation. In this study, we observed that E2/ERβ inhibited isoproterenol (ISO)-induced myocardial cell apoptosis, cytochrome *c* release and downstream apoptotic markers. Moreover, we found that E2/ERβ blocks ISO-induced apoptosis in H9c2 cells through the enhancement of calcineurin protein degradation through PI3K/Akt/MDM2 signaling pathway. Our results suggest that supplementation with estrogen and/or overexpression of estrogen receptor β gene may prove to be effective means to treat stress-induced myocardial damage.

## 1. Introduction

According to the statistics from the World Health Organization, heart disease is the most common cause of disease-related death worldwide and is markedly more common in men than in women. However, heart disease risk and incidence surges sharply in women with increasing age [1]. Estrogen, in the steroid hormones super-family, functions as the female sex hormone. Like all steroid hormones, estrogen can easily diffuse across the cell membrane and then translocate into cytosol to interact with its receptor. Two estrogen receptors, the estrogen receptor α (ER α) and the estrogen receptor beta (ER β), can interact with 17β-estradiol (E2). According to clinical reports, E2 is the most abundant and most active estrogen in women. Both ER α and ER β exist in the cardiomyocytes and E2 has been known to prevent development of heart disease via ER β [2,3,4,5,6]. E2/ER signaling is also known to modulate cAMP-PKA signaling/estrogen receptors (ERs) signaling interaction, to regulate Ca^2+^ influx and protect hearts.

Myocardial infarction, which results from cardiac ischemic injury, progresses to cardiac remodeling [7,8]. Accumulating evidence suggests that hypertrophy and apoptosis in cardiomyocytes contribute to the progression of heart failure and arrhythmias [7,9,10,11,12]. Previous reports show that estrogen is involved in modulating calcium-handling proteins, heart function, and blood flow; it reduces vascular inflammation and arrhythmias; prevents cardiac hypertrophy and myocardial cell apoptosis [13,14,15]. Protein phosphatase, such as PP2B (calcineurin) and type 1 phosphatase (PP1) are involved in cardiac hypertrophy, apoptosis, calcium influx, and heart failure. The expression levels and activity of PP1 as well as calcineurin are known to be elevated in failing human hearts [16]. It has been well established that dephosphorylation of phospholamban (PLB) by PP1 facilitates the binding of PLB to sarcoplasmic reticulum (SR) calcium ATPase (SERCA) to inhibit SERCA activity and Ca^2+^ uptake into SR for storage. Moreover, PKA phosphorylates and activates key proteins of the excitation-contraction coupling, such as L-type calcium channels (LTCC) as well as troponin I, a regulatory thin filament protein [17,18]. Regulation of cardiac LTCC function includes channel phosphorylation by protein kinases and dephosphorylation by phosphatases. Unregulated and steady increase in free Ca^2+^ in the cytosol leads to the activation of calcineurin and consequent apoptosis in susceptible cells which potentially leads to heart failure [19]. Overexpression of active calcineurin has been known to induce apoptosis through a mechanism which can be suppressed by Bcl-2 [20]. The pro-apoptotic protein Bcl2-associated death promotor Bad forms heterodimers with anti-apoptotic Bcl-2 and Bcl-XL, thereby accelerates cell death [21]. However, phosphorylation of the Bad by protein kinases such as Akt impairs its ability to bind with the anti-apoptotic proteins and inhibits apoptosis [22]. Calcineurine triggers apoptosis by either dephosphorylating NFAT to activate the subsequent transcription of apoptosis genes or by dephosphorylating Bad and thereby facilitating the binding of Bad to anti-apoptotic proteins. Isoproterenol (ISO) has been known to induce apoptosis of cardiac myocytes in vivo through β-adrenergic receptors. Previous experiments with calcineurin inhibitors and L-type Ca^2+^ channel antagonist show that ISO induces the apoptosis of cardiomyocytes with an increase in Ca^2+^ levels. ISO further reduces the phosphorylation of levels of Bad and triggers cytochrome *c* release [23].

However, the mechanisms of E2/ER β that suppress ISO-induced myocardial apoptosis are not fully understood [14], and the interaction of E2/ER β with phosphatase in the development of cardiac apoptosis is awaiting further investigation. Therefore, in this study we established a Tet-on ER β system in H9c2 myocardial cells and neonatal rat ventricular myocyte (NRVM) cells, to identify if E2/ER β inhibit ISO-induced myocardial cell apoptosis effects, and further investigated the roles of phosphatases (PP1 and PP2B) in the effect of E2/ER β.

## 2. Results

### 2.1. 17β-Estradiol (E2)/Estrogen Receptor Beta (ERβ) Inhibits Isoproterenol (ISO)-Induced Cellular Apoptosis in Tet-On ERβ H9c2 Myocardial Cells

**Figure 1:**
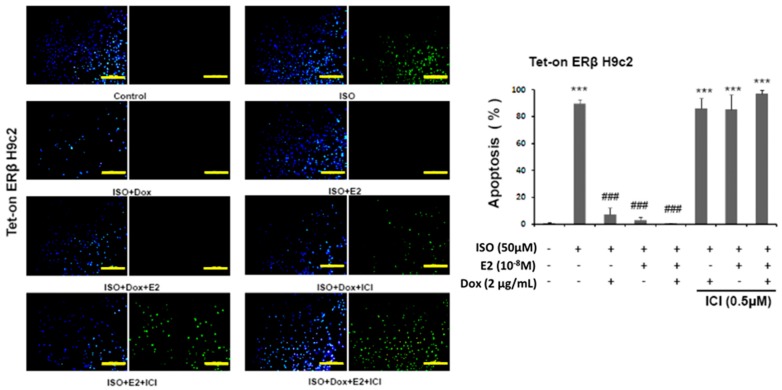
E2/ERβ inhibits ISO-induced cellular apoptosis in Tet-on ERβ H9c2 myocardial cells. Tet-on/ERα H9c2 cardiomyoblast cells were incubated with Dox (1 μg/mL) and E2 (10^−8^ M) in presence or absence of ISO (50 μM) and ICI (0.5 μM) for 24 h, then TUNEL and DAPI double-staining were performed. The images were detected by fluorescent microscope and the number of apoptotic nuclei was counted (Bar length = 100 μm). Mean ± S.D., *n* = 3. *** = *p* < 0.001 indicates significant difference with respect to the control group; ### = *p* < 0.001 indicates significant difference with respect to the ISO challenged group.

The results, as determined by TUNEL assay, reveal that pretreatment of estrogen (E2) and overexpression of estrogen receptor β (ERβ) effectively prevent ISO-induced cellular apoptosis. The number of apoptotic nuclei among the ISO administered cells was significantly higher when compared to the control group and the number was reduced in the presence of E2/ERβ. However, E2 and ERβ effects were inhibited with the pretreatment of 7α,17β-[9-[(4,4,5,5,5-Pentafluoropentyl)sulfinyl]nonyl]estra-1,3,5(10)-triene-3,17-diol (ICI), an estrogen receptor (ER) non-specific inhibitor that inhibits estrogen receptor α (ERα) and estrogen receptor β (ERβ). Therefore, the results show that E2/ERβ elicits a significant effect in suppressing the ISO-induced cellular apoptosis (Figure 1).

### 2.2. E2/ERβ Inhibits ISO-Induced Apoptosis Associated Caspase Activation and Cytochrome c Release in Tet-On ERβ H9c2 Myocardial Cells

**Figure 2:**
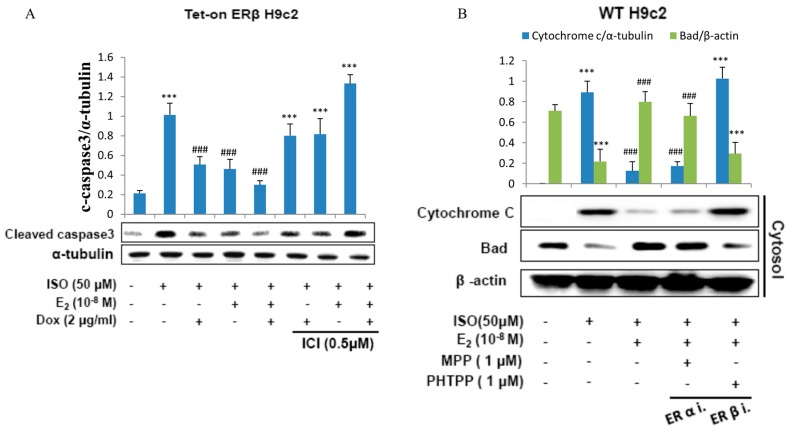
E2/ERβ inhibits ISO-induced mitochondria-dependent apoptosis in H9c2 myocardial cells. (**A**), Tet-on ERβ H9c2 cells were incubated with E2 (10^−8^ M), Dox (2 μg/mL), ICI (0.5 μM) in the presence of ISO (50 μM) for 24 h, then western blotting was performed. Cleaved caspase3 and α-tubulin were detected by Western blot. (**B**), H9c2 cells were incubated with E2 (10^−8^ M), MPP (1 μM), PHTPP (1 μM) in the presence of ISO (50 μM) for 24 h, then mitochondria isolation assay was performed. Cytochrome *c* and β-actin were detected by western blot. (*** = *p* < 0.001indicates significant difference with respect to the Control group; ### = *p* < 0.001 indicates significant difference with respect to the ISO challenged group).

To further confirm the effect of E2/ERβ on ISO induced apoptosis in H9c2 cardiomyoblast cells, proteins involved in the molecular events of apoptosis were analyzed by western blotting. The results show that ISO induced the apoptosis associated markers such as caspase-9, caspase-8, and caspase3; however administration of E2 or overexpression of ERβ effectively reduced the apoptotic proteins. Meanwhile, administration of ICI effectively blocked the effects of E2/ERβ (Figure 2A).

The western blot analysis further revealed that E2 and ERβ effectively prevented ISO-induced release of cytochome *c* into the cytoplasm. ISO treatment on H9c2 cells significant elevated the levels of cytoplasmic cytochome *c* however administration of E2 or overexpression of ERβ significantly reduced the levels of cytochome *c*. However, the ameliorating effect of E2 and ERβ was significantly inhibited when pretreated with estrogen receptor β inhibitor PHTPP and did not show much change when treated with the ERα inhibitor MPP (Figure 2B). The results therefore show that ERβ and not ERα is involved in attenuating ISO induced cytochrome *c* release.

### 2.3. E2/ERβ Attenuates ISO Induced Calcium Accumulation in H9c2 Cells

**Figure 3:**
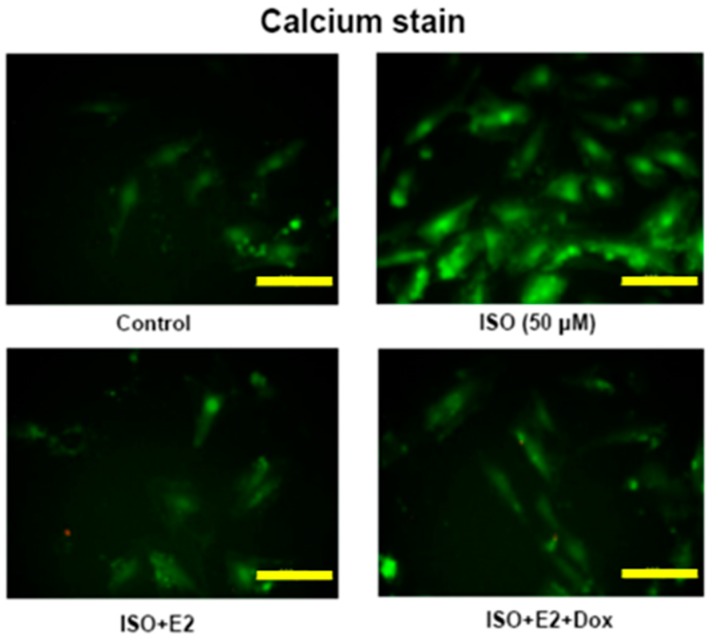
E2/ERβ attenuates ISO induced calcium accumulation in H9c2 cells. Tet-on ERβ H9c2 cells were incubated with E2 (10^−8^ M), Dox (2 μg/mL) in the presence of ISO (50 μM) for 24 h, then fluo-4AM calcium staining was performed. The images were detected by fluorescent microscope (Bar length = 10 μm).

To determine the effects of ISO on calcium accumulation in H9c2 cells the cells were stained by Fluo-4 AM. The ISO administered cells showed high levels of calcium accumulation as seen from the intensity of the Fluo-4 AM stain. However, in the E2 treated H9c2 cells or in E2 treated cells over-expressing ERβ the intensity of the stain reduced drastically, signifying the inhibitory effect of E2/ERβ ion ISO induced calcium accumulation (Figure 3).

### 2.4. Calcineurin Plays an Important Role in ISO-Induced Cellular Apoptosis Signaling

**Figure 4:**
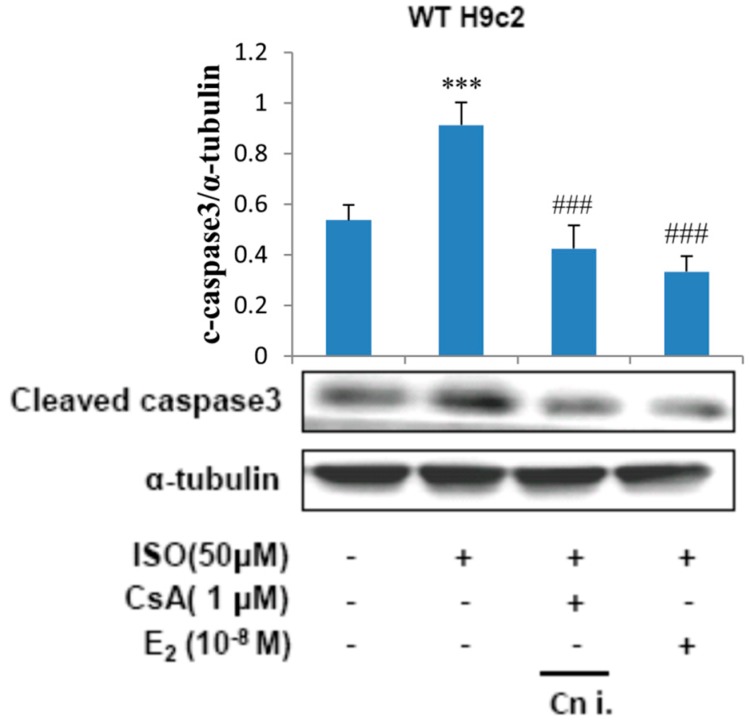
Calcineurin plays an important role in isoproterenol-induced cellular apoptosis signaling. H9c2 cells were incubated with E2 (10^−8^ M), CsA (1 μM) in the presence of ISO (50 μM) for 24 h, then western blotting was performed. Cleaved caspase3 and α-tubulin were detected by western blot. (*** = *p* < 0.001 indicates significant difference with respect to the control group; ### = *p* < 0.001 indicates significant difference with respect to the ISO challenged group).

To determine the involvement of calcineurin in the apoptotic effects induced by ISO, H9c2 cells were incubated with E2 (10^−8^ M) or calcineurin inhibitor CsA (1 μM) in the presence of ISO (50 μM) for 24 h and the respective total protein lysate was analyzed by western blotting. The levels of cleaved caspase3 was found to increase when treated with ISO, treatment with CsA however suppressed the effect of ISO on caspase3 levels. Therefore, the results reveal that the apoptosis induced by ISO is mediated through calcineurin. Treatment with E2 was effective in inhibiting the calcineurin mediated caspase activation induced by ISO (Figure 4).

### 2.5. E2 Enhances Calcineurin Protein Degradation via Estrogen Receptor β

**Figure 5:**
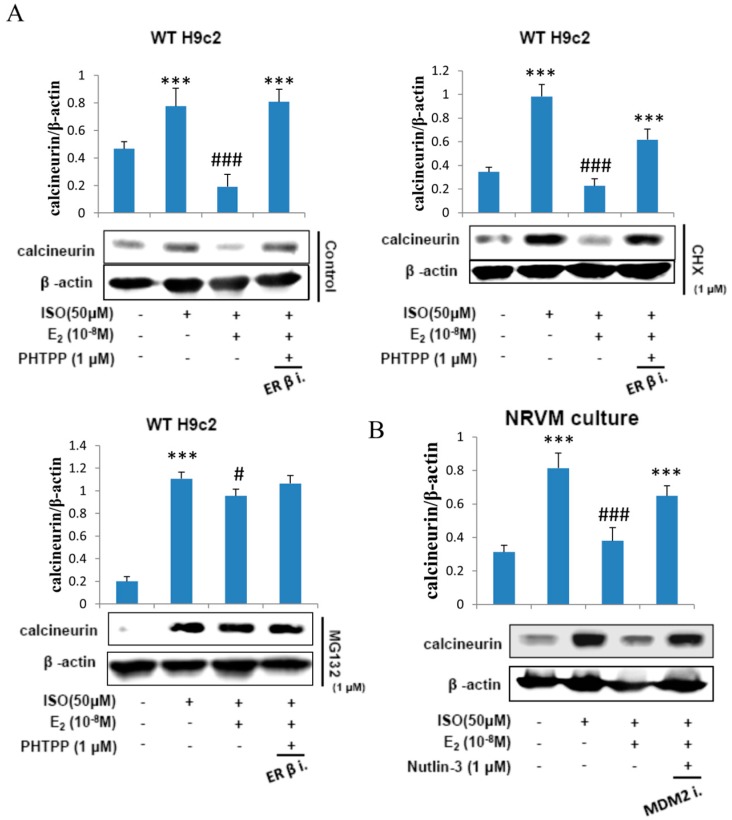
E2 enhances calcineurin protein degradation via estrogen receptor β. (**A**) H9c2 cells were incubated with E2 (10^−8^ M), ISO (50 μM), PHTPP (1 μM) in the presence of protein synthesis inhibitor cycloheximide (1 μM) and proteasome inhibitor MG132 (1 μM) for 24 h, then western blotting was performed. Calcineurin and β-actin were detected by western blot. (**B**) NRVM cells were incubated with E2 (10^-8^ M), Nutlin-3 (1 μM) in the presence of ISO (50 μM) for 24 h, then western blotting was performed. Calcineurin and β-actin were detected by western blot. (*** = *p* < 0.001 indicates significant difference with respect to the Control group; (# = *p* < 0.05 and ### = *p* < 0.001 indicate significant differences with respect to the ISO challenged group).

H9c2 cells were administered with ISO (50 μM), treated with E2 (10^−8^ M), ERβ inhibitor PHTPP (1 μM) in the presence of protein synthesis inhibitor cycloheximide (1 μM) and then in proteasome inhibitor MG132 (1 μM) for 24 h and the respective protein extracts were analyzed by western blotting. The data revealed that E2 treatment inhibited the effects of ISO on calcineurin but the inhibitory effect was attenuated when treated with PHTPP. Cyclohexamide did not have any effect on the modulations in calcineurin level inflicted by ISO or by E2. However, in the presence of the proteasome inhibitor MG132 the E2 triggered suppression of ISO induced calcineurin was completely blocked. The data therefore show that E2 mediated suppression in calcineurin level involves proteosomal degradation of calcineurin protein (Figure 5A).

Furthermore, to determine whether MDM2 plays a role in calcineurin degradation NRVM cells were administered with ISO (50 μM) and incubated with E2 (10^−8^ M) and MDM2 inhibitor Nutlin-3 (1 μM) for 24 h. The western blot analysis on the total protein extracts show that the E2/ERβ induced calcineurin protein degradation in ISO-induced apoptosis is MDM2 dependent (Figure 5B).

### 2.6. E2/ERβ Enhances Calcineurin Protein Degradation via PI3K/Akt/MDM2 Signaling

**Figure 6:**
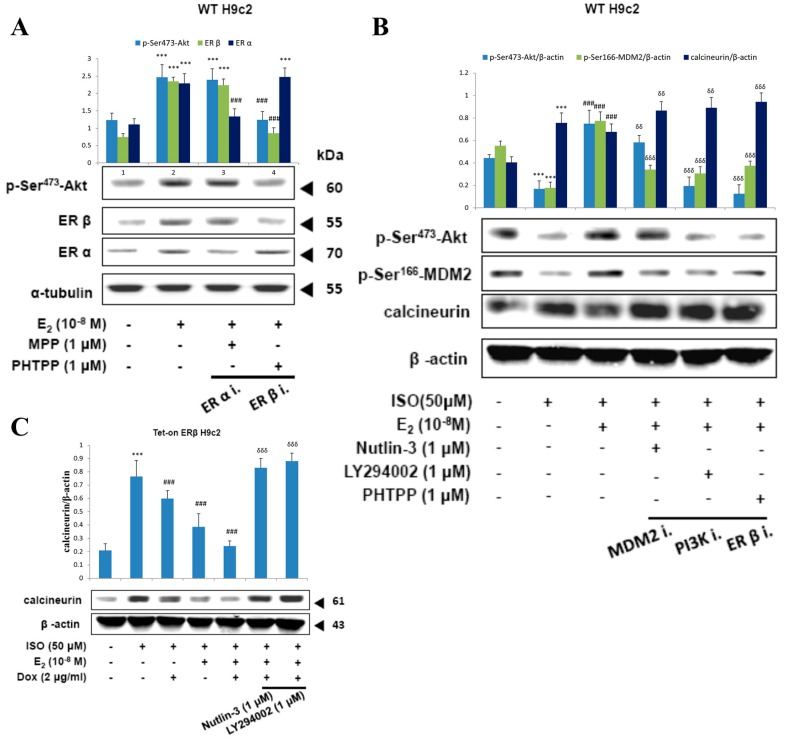
E2/ERβ enhances calcineurin protein degradation via PI3K/Akt/MDM2 signaling. (**A**), H9c2 cells were incubated with MPP (1 μM), PHTPP (1 μM) in the presence of E2 (10^−8^ M) for 24 h, then western blotting was performed. *p*-Akt (Ser473), ERα, ERβ, and α-tubulin were detected by western blot. (**B**), H9c2 cells were incubated with E2 (10^−8^ M), MDM2 inhibitor Nutlin-3 (1 μM), PI3K inhibitor LY294002 (1 μM), PHTPP (1 μM) in the presence of ISO (50 μM) for 24 h, then western blotting was performed. *p*-Akt (Ser473), *p*-MDM2 (Ser166), calcineurin and β-actin were detected by Western blot. (**C**), Tet-on ERβ H9c2 cells were incubated with E2 (10^−8^ M), Dox (2 μg/mL), Nutlin-3 (1 μM), LY294002 (1 μM) in the presence of ISO (50 μM) for 24 h, then western blotting was performed. Calcineurin and β-actin were detected by Western blot. (*** = *p* < 0.001indicates significant difference with respect to the Control group; ### = *p* < 0.001 indicates significant difference with respect to the ISO challenged group, δδ = *p* < 0.01 and δδδ = *p* < 0.001 indicate significant differences with respect to the E2 treated group).

Treatment with E2 in H9c2 cells showed upregulation of p-ser^473^-Akt levels however, inhibition of ERβ with PHTPP caused a reversion in the elevated levels of p-ser^473^-Akt to normal levels, indicating the involvement of ERβ in E2 induced Akt activation. Meanwhile, treatment with ERα inhibitor MPP caused no effect on the E2 induced Akt levels. Therefore, the data show that E2 induced Akt activation only through ERβ (Figure 6A).

Administration of ISO suppressed the levels of p-ser473-Akt and p-ser166-MDM2 correlated with an enhancement in calcineurin levels. As expected, the levels were reverted to normal levels when treated with E2 however, in the presence of MDM2 inhibitor Nutlin-3, the effect of E2 on the levels of p-ser166-MDM2 and calcineurin was attenuated and in the presence of PI3K inhibitor LY294002 and in the presence of ERβ inhibitor the effect of E2 on p-ser473-Akt, p-ser166-MDM2, and calcineurin were all reverted. The results therefore show that E2/ERβ acts through PI3K to activate Akt which in turn activates MDM2 and calcineurin degradation to suppress ISO induced apoptosis (Figure 6B).

Further to clarify the effective role of ERβ in the PI3K/Akt/MDM2 mediated calcineurin degradation, ERβ was overexpressed by Doxycycline in Tet-on/ERα H9c2 cells. Overexpression of ERβ enhanced calcineurin degradation and the synergistic effect of ERβ with E2 showed further enhancement in calcineurin treatment. However, the effects of ERβ and E2 were reverted on inhibiting MDM2 and PI3K. Therefore, the results confirm that E2/ERβ enhance the calcineurin degradation via PI3K/Akt/MDM2 signaling.

## 3. Discussion

The sympathetic nervous system (SNS) plays an integral role in regulating cardiac function. However, evidence suggests that enhanced SNS activation can have harmful effects on hearts, resulting in heart failure [24,25]. Hence, regulation of the SNS with the administration of β-adrenergic receptor (β-AR) blockers in cardiomyopathy represents a potential therapeutic strategy. In clinical observations, therapeutic interventions using β-AR blockers not only efficiently improve the cardiac contractility but also greatly improve the prognosis of heart failure [26,27].

Pathophysiological stimuli such as neurohumoral activation (*viz*. angiotensin II (ANG II) and β-adrenoceptor (β-AR) stimulation with ISO), hypertension and diabetic cardiomyopathy cause cardiac hypertrophy, apoptosis, and heart failure [28,29]. Cardiomyocyte apoptosis are observed to be elevated in severe hypertrophic conditions and intensifies the adverse outcomes of LV hypertrophy associated in terms of reduction in the cardiac contraction and function [30]. Previous reports indicated that activation of β-AR by elevated plasma NE and ISO causes serious cardiac cell apoptosis in vivo and in vitro [31,32,33]. In the present study, we found that ISO induces myocardial apoptosis through calcineurin. However, E2/ERβ effectively attenuated the ISO-induced myocardial apoptosis as determined by TUNEL assay and western blot analysis on the apoptotic associated proteins. Previous studies point out that estrogen can enhance calcineurin protein degradation via 26S proteasome. Calcineurin degradation has been shown previously to depend on the specific E3 ubiquitin ligase atrogin1, which associates with calcineurin and catalyzes CnA ubiquitination [34,35,36,37]. Previous studies show that TAC induces a significant decline in the expression of atrogin1, MDM2, and MuRF1 in placebo-treated mice. E2 replacement had no effect on the TAC-induced decrease in atrogin1 expression but restored the expression of both MDM2 and MuRF1 to levels similar to shams [34]. In the present study, we found that E2/ERβ can enhance calcineurin protein degradation by MDM2 E3 ligase, as observed in the present study by western blotting.

It is known that menopause and ovariectomy increases the risk of ischemic heart disease that involves loss of cardiomyocytes with increase in apoptosis [38,39,40]. Extrinsic apoptotic pathway is believed to be one of the major mechanisms of cell death in E2 deficient or ovariectomized rats [41]. It is often initiated by Fas ligand or tumor necrosis factor-alpha (TNFα). Activation of Fas-associated death domain (FADD) either by Fas or TNFα prompts the activation of caspase-8 which results in caspase3 cleavage and activation. A second type of apoptosis that is mitochondria-dependent apoptosis also contributes to the cardiomyocyte loss and is characterized by the mitochondrial release of an array of pro-apoptotic factors such as Bax and Bad. When the pro-apoptotic factors are not sufficiently neutralized by the anti-apoptotic proteins such as Bcl-2, they trigger cytochrome *c* which activates capase-9 which can in turn activate caspase-8. Mitochondria-mediated apoptosis is also significantly increased in the ovariectomized rats [41].

Previous reports show that estrogen administration modulates TNFα and TNFα receptors and attenuated apoptosis associated with ischemia/reperfusion injury [42]. TNFα, a predominant cytokine that is elevated in estrogen deficient rats is involved in inflammation, apoptosis, and enhanced vascular remodeling. In motoneurons, enhanced activation of ER provides neuroprotection by attenuating the TNFα mediated apoptosis [43]. E2 supplementation in Langendorff-perfused rat heart model of stop-flow ischemia resulted in the suppression of mitochondrial damage and cellular apoptosis and provided cardio-protection against ischemic damage [44]. Investigation on the effect of E2 supplementation in ovariectomized rats show that E2 effectively attenuates extrinsic as well as the intrinsic apoptosis in the heart [45]. While E2 administration can regulate both the major apoptotic pathways, ISO induced apoptosis originating from β-AR is generally known to be the outcome of calcineurin activation and mitochondrial dependent apoptosis [23]. ISO activated calcineurin induces apoptosis by dephosphorylating Bad and thereby destroy the mitochondrial membrane potential and induce apoptosis. The activated calcineurin also may induce hypertrophy either by activating NFAT-3 [46]. Although many other studies have indicated other mechanisms induced by ISO, they have not superseded the involvement of calcineurin activation and Bad dephosphorylation in ISO-induced apoptosis [47,48,49,50]. In this present study, we focus on the cardiac apoptosis induced by ISO and checked the elevation of Bad and cytochrome *c* as an indicator of apoptosis induced by ISO. 

Further, previous studies indicated that PI3K-Akt pathway is involved in the anti-apoptotic effects of certain stimuli and plays central role in cellular survival in many different cell types [51]. PI3K-Akt pathway is estrogen receptor β-dependent in ERβ knockout mice [52]. In addition, MDM2 activation is protein kinase B/Akt-dependent phosphorylation [53]. Besides, the cell apoptosis is downregulation of the PI3K-Akt-MDM2 pathway activation [54].

ERβ has been known to provide cardio protection in female mice hearts against ischemia though the activation of the PI3K/Akt pathway. Estrogens are known to upregulate SUR2A, a regulatory subunit of sarcolemmal K_ATP_ channels that binds to inward rectifier Kir6.2 to form cardiac sarcolemmal ATP-sensitive K^+^ channels. Upregulation of SUR2A increases the number of K_ATP_ channels and thereby enhances the cardiac resistance to stress [55,56]. In the present study, the activation of PI3K-Akt may persuade to draw similarities to the effect of ER on the β-AR activation associated apoptosis and the protective effects of SUR2A against ischemia related effects [56,57]. However, careful investigations should be carried out to draw further conclusions.

In the present study, we found that PI3K-Akt-MDM2 signaling activation is estrogen receptor β-dependent and enhances calcineurin protein degradation. Moreover, E2/ERβ induced calcineurin protein degradation further inhibits ISO-induced myocardial apoptosis.

The cardiovascular disease incidence and mortality among premenopausal women is remarkably low while being significantly high in menopausal women, this signifies the female hormone estrogen as a cardio-protector. In contrary, reports from the Women’s Health Initiative (WHI) advocates that estrogen therapy in postmenopausal women does not provide any relief against the onset of cardiomyopathy [58]. However, the previous study is confined to vasculature and differs from the phenomenon investigated in this study. Further, it is evident that estrogen ameliorates atherosclerosis effects by lowering low-density lipoproteins and inflammatory processes in the vasculature [59,60]. Moreover, our findings suggest that estrogen potentially prevents the development of early atherosclerotic lesions prior to the onset of atherosclerosis effects.

Conditions such as diabetes and hypertension, which are associated with ischemic conditions, generally trigger cardiac hypertrophy and apoptosis that correlates with the increase in inflammatory factors. However, hyperglycemia in spontaneously hypertensive animal models amplifies the pathological apoptosis and hypertrophy without much difference in the mediators of inflammation. Therefore, it would be interesting to determine the effects of E2/ERβ with reference to the effects of ISO in any pathological conditions such as diabetes that promote various metabolic intermediates to encourage pathological events such as apoptosis [61].

In the present study, we investigated the cardioprotective effects and mechanisms provided by E2 and ERβ in myocardiac cells exposed to β-adrenergic receptor agonist, ISO. The present study clearly shows that the cardioprotective effects and mechanisms of E2 and ERβ involve in mediating calcineurin activity and mitochondrial stability of myocardial cells. In conclusion, we found that activation of estrogen receptor β by E2 significantly inhibits cardiac apoptosis and enhances calcineurin protein degradation by PI3K-Akt-MDM2 signaling activation to inhibit ISO-induced myocardial cell apoptosis.

## 4. Material and Methods

### 4.1. Cell Culture

The rat cardiomyoblast cell line-H9c2 (CRL-1446) was purchased from American Type Culture Collection (ATCC Cell Biology Collection, Manassas, VA, USA). Cell cultures were maintained in DMEM (supplemented with 10% Cosmic Calf Serum (CCS), 1% antibiotic-antimycotic, 1.5 g/L of sodium bicarbonate and 3.5 g/L of Glucose) at 37 °C in 5% CO_2_ in a humidified incubator.

### 4.2. Cardiomyocyte Culture

Neonatal cardiomyocytes were isolated and cultured from one-day-old newborn Sprague-Dawley rats (BioLASCO, Taipei, Taiwan) using the commercially available Neonatal Cardiomyocyte Isolation Kit following the manufacturer’s instructions (Cellutron Life Technology, Highland Park, NJ, USA). Ventricular cardiomyocytes were pooled and were cultured in NS medium (Cellutron Life Technology) with 10% fetal bovine serum.

### 4.3. Construct Tet-On Gene Expression System

Tet-on Gene Expression System is one of methods that use two different expression plasmids to cooperatively control gene expression. Plasmid pTet-on, constitutively expressed rtTA protein which binds to the promoter of pTRE plasmid and activates it along with Dox or Tet. The pTRE2-ERβ response plasmid which constructed with the splicing of pTRE2hyg-Luc plasmid and ERβ cDNA between the 5′ BamHI and 3′ SalI restriction site such that ERβ is expressed under the control of tetracycline-response element (TRE). The transfection and clonal selection were performed as mentioned following methods mentioned in our previous report [62].

### 4.4. Western-Blot Analysis

Western blot analysis was performed following previous reports with slight modification [63]. Cultured cells were washed with cold PBS and then lysed by spending in lysis buffer [(50 mM Tris, pH 7.5, 0.5 M NaCl, 1.0 mM EDTA, pH 7.5, 10% glycerol, 1 mM BME, 1% IGEPAL-630, and proteinase inhibitor cocktail (Roche Molecular Biochemicals, Upper Bavaria, Germany)]. After 30 min incubation on ice, the contents were centrifuged at 12,000× *g* for 15 min at 4 °C, and the protein extract was collected from the supernatant and the total protein content was quantified by Bradford method. The protein samples (40 μg) were separated by electrophoresis on a SDS polyacrylamide gel and transferred onto a PVDF membrane (Millipore, Belford, MA, USA). The membranes were washed and blocked in blocking buffer (5% non-fat dry milk, 20 mM Tris–HCl, pH 7.6, 150 mM NaCl, and 0.1% Tween 20) for 1 h and then the membranes were incubated with diluted primary overnight on a shaker at 4 °C. Membranes hybridized with the primary antibodies were further incubated with appropriate horseradish peroxidase-linked secondary antibodies and the results were recorded on a chemiluminescence documentation system (Image quant, LAS4000 mini, GE Healthcare Life Sciences, Pittsburgh, PA, USA).

### 4.5. 4',6-Diamidino-2-phenylindole DAPI Staining and In Situ Terminal Deoxynucleotide Transferase-Mediated dUTP Nick End-Labeling TUNEL Assay

Cells were grown in 12-well plates and after treatments they were fixed with 4% paraformaldehyde treatment for 30 min. The cells were permeabilized with 0.1% TWEEN 20 and were then incubated with in situ terminal deoxynucleotide transferase-mediated dUTP nick end-labeling (TUNEL) reagent from the In Situ Cell Death Detection Kit, Fluorescein (Roche, Basel, Switzerland), as per manufacturer’s instructions. Under a fluorescence microscopy (Olympus, Tokyo, Japan), the nuclei were illuminated in blue with DAPI staining and the TUNEL positive nuclei were illuminated in green. The number of TUNEL positive cells was counted from three independent experiments which were then averaged and statistically analyzed.

### 4.6. Intercellular Calcium Staining

Tet-on ERβ H9c2 cells cultured on 6 cm^2^ dishes were incubated for 20 min with 2 μM of Fluo-4 AM. After incubation, the cells were washed thrice with calcium containing HBSS solution and then incubated in calcium containing HBSS solution for 15 s. The Fluo-4 AM fluorescence images of the cells was recorded with a laser scanning confocal microscope (Leica Microsystems, Wetzlar, Germany) through a 515 nm long pass emission filter.

### 4.7. Statistical Analysis

The data were collected and the mean of three independent repeats were statistically evaluated using the paired, two-tailed *t*-test. The data is represented as means ± S.D.
